# Can omic tools help generate alternative newer sources of edible seed oil?

**DOI:** 10.1002/pld3.399

**Published:** 2022-06-07

**Authors:** Parimalan Rangan, Rasna Maurya, Shivani Singh

**Affiliations:** ^1^ Division of Genomic Resources ICAR‐National Bureau of Plant Genetic Resources New Delhi‐12 India

**Keywords:** acetyl‐CoA carboxylase, cytosolic TAG biosynthesis, de novo TAG biosynthesis, gene regulation, lipid droplet, next generation sequencing, oilseed, phosphatidylcholine derived pathway, triacylglycerol

## Abstract

There are three pathways for triacylglycerol (TAG) biosynthesis: De novo TAG biosynthesis, phosphatidylcholine‐derived biosynthesis, and cytosolic TAG biosynthesis. Variability in fatty acid composition is mainly associated with phosphatidylcholine‐derived TAG pathway. Mobilization of TAG‐formed through cytosolic pathway into lipid droplets is yet unknown. There are multiple regulatory checkpoints starting from acetyl‐CoA carboxylase to the lipid droplet biogenesis in TAG biosynthesis. Although a primary metabolism, only a few species synthesize oil in seeds for storage, and less than 10 species are commercially exploited. To meet out the growing demand for oil, diversifying into newer sources is the only choice left. The present review highlights the potential strategies targeting species like *Azadirachta*, *Callophyllum*, *Madhuca*, *Moringa*, *Pongamia*, *Ricinus*, and *Simarouba*, which are not being used for eating but are otherwise high yielding (ranging from 1.5 to 20 tons per hectare) with seeds having a high oil content (40–60%). Additionally, understanding the toxin biosynthesis in *Ricinus* and *Simarouba* would be useful in developing toxin‐free oil plants. Realization of the importance of cell cultures as “oil factories” is not too far into the future and would soon be a commercially viable option for producing oils in vitro, round the clock.

## Highlight

- Newer alternative sources like cell cultures and high yielding (nonedible) species with high oil content are commercially viable targets to engineer in producing edible‐grade oil using the omics tool.

**Abbreviations**
ACCase: acetyl‐CoA carboxylase
DGAT: diacylglycerol acyltransferase
LD: lipid droplet
PC: phosphatidylcholine
WRI: wrinkled

## 1 INTRODUCTION

Seeds are sink tissues containing nutritional reserves such as carbohydrates (as starch or hemicellulose), proteins, and fats or oils (in different proportions). They are utilized during the germination process for initial establishment and growth of a plant. Of the three types of reserves, carbon density of fats and oils are nearly double when compared with carbohydrates (Lüttge, 2013). Due to their energy‐dense nature, the construction cost of fats and oils is the highest among the three reserves, and nearly double the cost of carbohydrate construction. Carbon in its organic form is essential for all the energy storage and transfer processes (Mooney, 1972). It is these sink tissues, seeds of cereals or pulses or oilseeds, that act as the seeds of nutrition and source of energy for most of the global population (Heiser, 1990; Smil, 2017). In short, humans domesticated the present‐day crops, and through culturing these crops, humans traveled the path of civilization. Roughly, 30% of the calorie requirements (through dietary intake) is contributed through fats and oils (Mustafa & Iqbal, 2021).

Plant oils or vegetable oils are energy‐rich long chain hydrocarbons and provide maximal calorie equivalents compared with carbohydrates or proteins. There is an upsurge in the demand for vegetable oils due to their both edible and nonedible applications including biofuel and other industrial applications that have caused increase in prices of vegetable oils (Lu et al., 2011), and a balance between the demand and supply would stabilize the oil economy. Alternative newer sources of plants or crops to add to the oil market are thus a dire need to cater to the growing demand. Additionally, for edible purposes, a few nonedible (due to presence of certain alkaloids or compounds) seed oil plants could be modified appropriately using precise gen(om)e editing tools to make them edible types. The present review gives an overview of oil biosynthesis with potential newer plant oil sources and strategies to make them edible by removing their non‐edibility factors, genetically.

## 2 TRIACYLGLYCEROL BIOSYNTHESIS—AN OVERVIEW

Seeds are the major storage organs of plant lipids (oils). Their oils contribute to 60–80% of the seeds' dry weight (Ohlrogge & Browse, 1995; Voelker & Kinney, 2001). Triacylglyceride (TAG) biosynthetic pathway is comparatively conserved but varies depending on the species (in the last few steps of the TAG biosynthesis) and common to most of the plant tissues. However, there is a large diversity among species regarding the acyl chain length and the degree and position of unsaturation. These diverse factors stabilize the oil across time and temperature (Voelker & Kinney, 2001). There are three major pathways for lipid biosynthesis, namely, de novo TAG biosynthesis, phosphatidylcholine (PC)‐derived TAG biosynthesis, and cytosolic TAG biosynthesis. Of the three pathways, de novo and cytosolic pathways are acyl‐CoA‐dependent whereas the PC‐derived pathway is acyl‐CoA‐independent (Bates & Browse, 2012; Dahlqvist et al., 2000; Lu et al., 2009; Ohlrogge & Browse, 1995; Saha et al., 2006; Weselake et al., 2009). The chloroplast (in non‐green tissues, chromo‐ or leuco‐plasts) is the major organelle that synthesizes fatty acids (FAs) and transports them to endoplasmic reticulum (ER) where TAG formation occurs (Domínguez & Cejudo, 2021; Hölzl & Dörmann, 2019; Lüttge, 2013). The formed TAGs accumulate between the bilayered phospholipid membrane of the ER, thereby forming a lipid lens. This further grows and forms into a mature lipid droplet (LD) consisting of TAGs within the phospholipid monolayer that buds off from the ER into the cytoplasm and is known as oil bodies or oleosomes or spherosomes, which generally range in the sizes of 0.5 to 2.0 μm but can be up to 20 μm (Chapman et al., 2019, 2012; Lüttge, 2013; Pyc et al., 2017; Siloto et al., 2006). In a similar fashion to lipid biosynthesis pathway, LD biogenesis is conserved across most organisms, while the proteins like SEIPIN and oleosins vary specifically at the species level, and are associated mainly with the size of the LD (Cai et al., 2015; Chapman et al., 2019, 2012; Pyc et al., 2017; Siloto et al., 2006). However, nonseed LDs are oleosin‐independent (Chapman et al., 2012; Siloto et al., 2006).

**FIGURE 1:**
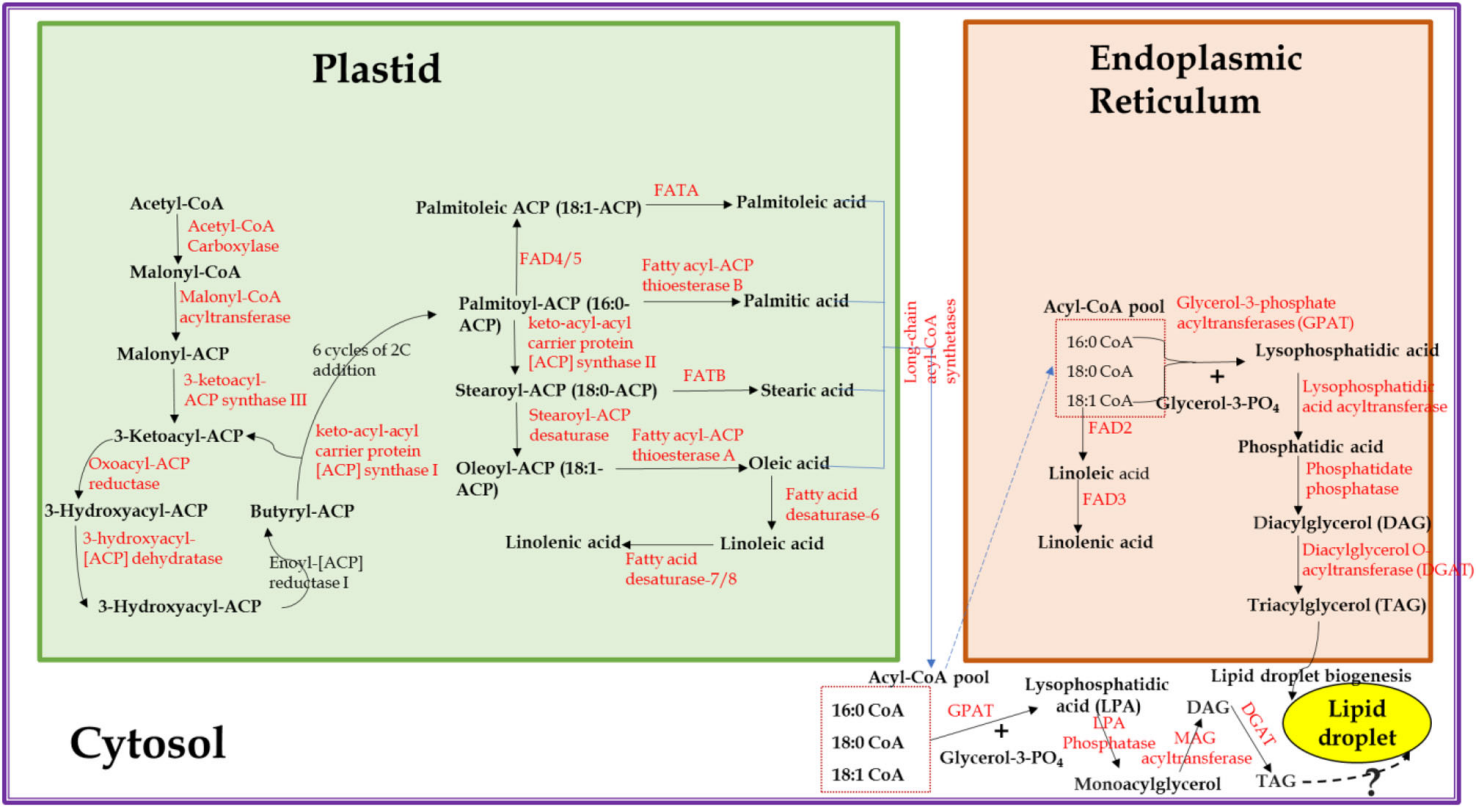
De novo and cytosolic triacylglycerol biosynthesis

De novo TAG biosynthesis, also known as the Kennedy pathway, was illustrated in 1961 (Kennedy, 1961), with step‐wise evidence reported earlier, and constitutes the formation of TAGs from glycerophosphate (Weiss et al., 1960; Weiss & Kennedy, 1956). There are numerous studies that describe de novo TAG biosynthesis (Bates & Browse, 2012; Lu et al., 2011; Ohlrogge & Browse, 1995; Weselake et al., 2009). In brief, they can be grouped into three steps, namely, FA synthesis in plastids, acyl‐CoA pool formation in the cytosol, and TAG formation in ER that consequently forms as LDs in cytosol (Figure 1). FA synthesis in plastid is initiated through the conversion of acetyl‐CoA to malonyl‐CoA through acetyl‐CoA carboxylase (ACCase) and in a series of steps, free FAs are formed (for details, please refer the green‐shaded portion of Figure 1). Formed free FAs are then converted into their acyl‐CoA pools in the cytosol through the catalytic action of long‐chain acyl‐CoA synthetases (LCAS). In the third group of steps, the acyl‐CoA pools are transported to the ER and upon acylation of glycerol‐3‐phosphate at the first and second positions form phosphatidic acid (PA). Dephosphorylation of PA through phosphatase enzyme forms diacylglycerol (DAG) which is converted to TAG (for details, please refer the brown‐shaded portion of Figure 1). This third group of steps within the de novo TAG biosynthesis is referred to as the Kennedy pathway (Bates & Browse, 2012; Kennedy, 1961).

Besides de novo TAG biosynthesis, TAGs are also formed through the formation of DAG from PC, a membrane lipid, and this alternate route is known as PC‐derived or acyl‐CoA‐independent TAG biosynthesis (Bates & Browse, 2012; Dahlqvist et al., 2000; Lu et al., 2009). The oleoyl‐CoA (18:1) after being incorporated in phosphatidylcholine (PC) undergoes polyunsaturation to form 18:2 and 18:3 through the catalytic action of FAD2 and FAD3 desaturases in the ER (Stymne & Appelqvist, 1978). This PC‐derived route diversifies the lipid biosynthesis through various enzymatic reactions like hydroxylation, epoxylation, and acetylation on PC and hence modified PC is involved in the formation of DAGs (Lu et al., 2009 and citations thereof). Inter‐conversion between two molecules of DAG and monoacylglycerol (MAG) + TAG does occur through transacylation reaction in an acyl‐CoA‐independent manner (Stobart et al., 1997).

For TAG biosynthesis, in addition to the two pathways, de novo (acyl‐CoA‐dependent) and PC‐derived (acyl‐CoA‐independent), both accomplishing in the plastid and the ER, a third route (acyl‐CoA‐dependent) in the cytosol is reported (Saha et al., 2006). The cytosolic route differs from the de novo TAG biosynthesis in the third group of steps (TAG formation in ER). The first two groups of steps of the de novo TAG biosynthesis, FA synthesis in plastids and acyl‐CoA pool formation in the cytosol are common. In the cytosolic route, lysophosphatidic acid (LPA) is formed from the glycerol‐3phosphate in cytosol, and the formed LPA is converted to MAG through the catalytic action of cytosolic LPA phosphatase (Shekar et al., 2002). The enzyme MAG acyltransferase catalyzes the conversion of MAG to DAG, utilizing acyl‐CoA (Tumaney et al., 2001). The final conversion of DAG to TAG is catalyzed through the cytosolic DAG acyltransferase (DGAT), utilizing acyl‐CoA (Saha et al., 2006). We call this cytosolic (also acyl‐CoA‐dependent, like the de novo pathway) TAG biosynthesis pathway as the “Rajasekharan pathway.” Evidence for the cytosolic LD biogenesis of the TAG molecules synthesized in the cytosol, which requires the phospholipid monolayer and the LD‐associated proteins—oleosins, caleosins, and steroleosins (Chapman et al., 2019, 2012), is unknown.

## 3 REGULATION OF TAG BIOSYNTHESIS

The TAG biosynthesis pathway is regulated in the steps involved in the conversion point of photosynthates (glucose, sucrose, and their derivatives) to FA, and also in the steps between FA and TAG formation (Guschina et al., 2014; Zhai et al., 2021). The regulatory mechanisms can be grouped into key factors such as transcriptional and post‐translational factors. Metabolites, hormones, long noncoding RNAs (lncRNA), miRNAs, and stress are the key interactive factors that modulate TAG formation (Batsale et al., 2021; Coleman & Lee, 2004). In addition, LD biogenesis is also regulated dynamically through the LD‐associated proteins (Chapman et al., 2019; Gidda et al., 2016; Pagac et al., 2016; Shi et al., 2013). ACCase is identified as playing a key regulatory role in TAG biosynthesis (Post‐Beittenmiller et al., 1992; Weselake et al., 2009), although it is regulated through the presence of 2‐oxoglutarate (Zhai et al., 2021). Modifications of FA through hydroxylation, epoxylation and acetylation process comes at the cost of nearly 50% reduction of the quantum in de novo TAG biosynthesis and are regulated by ACCase (Bates et al., 2014). Recent updates on the TAG biosynthesis regulation are detailed here.

Key transcription factors Wrinkled1 (WRI1), Leafy cotyledon (LEC1 and 2), abscisic acid insensitive (ABI3) are known to regulate TAG biosynthesis (Lu et al., 2011; Weselake et al., 2009). Regulation of TAG biosynthesis at the transcriptional level is mediated through three transcription factors: GmZF392, GmZF351, and GmNFYA (Lu et al., 2021). Trehalose‐6‐phosphate is reported to act as a signal molecule for SnRK1 to phosphorylate the transcription factor WRI1 for downstream regulatory process and also regulate DGAT1, associated in TAG biosynthesis (Guschina et al., 2014; Lu et al., 2011; Zhai et al., 2021). WRI1 and 2 regulate the genes and their products that are involved in the FA synthesis part of TAG biosynthesis (Behera et al., 2021; Focks & Benning, 1998). In addition to phosphorylation‐mediated regulation of DGAT1 post‐transcriptionally, miRNA‐mediated regulation of the transcripts of *DGAT1* is regulated in multiple ways (SnRK1, miRNA, and phosphorylation of the DGAT1 itself) for the conversion of DAG to TAG (Wang et al., 2021; Weselake et al., 2009; Zhai et al., 2021; Zhang et al., 2021). Recent reports underscore the importance of lncRNA (Chen et al., 2021), and miRNA (Wang et al., 2021) in regulating the genes associated with TAG biosynthesis.

## 4 UNUSUAL FAs

The five FAs palmitic (16:0), stearic (18:0), oleic (18:1), linoleic (18:2), and linolenic (18:3) are the most common ones constituting more than 90% of the acyl chains found in the TAG and membrane lipids (Cahoon & Li‐Beisson, 2020; Ohlrogge & Browse, 1995; Voelker & Kinney, 2001). Besides, there are unusual FAs (uFA, more than 450) specific to a certain group of plants and are mostly limited to seed TAGs and are likely to be associated with speciation events and therefore species‐specific (Cahoon & Li‐Beisson, 2020). uFAs are FAs containing either less than 16 or more than 18 carbon atoms, or odd‐numbered FAs, or with variable double bond positions other than that of the common FAs, or modifications of the FAs (mFAs) through hydroxylation, epoxylation, acetylation, and other similar reactions (Cahoon & Li‐Beisson, 2020; Diedrich & Henschel, 1990; Lu et al., 2009; Takagi & Itabashi, 1982; van de Loo et al., 2018; Voelker & Kinney, 2001). In castor, nearly 90% of the unsaturated FA are constituted with hydroxylated FA (18:1‐OH) with the oil content up to 50% by weight (Mubofu, 2016). Hydroxylation events in transgenic *Arabidopsis* is reported to inhibit (nearly 50%) the de novo TAG biosynthesis. However, when regulated through ACCase (Bates et al., 2014; Bates & Browse, 2011), as accomplished by the researchers, the inhibition was overcome. This suggests that the regulatory mechanism of ACCase genes from a different species are very different, underscoring the importance of sequence associated post‐translational modifications in TAG pathway regulation.

Members of Araceae, Lauraceae, Lythraceae, and Ulmaceae are known to contain acyl chains of less than 16 carbon atoms, and Brassicaceae, Cruciferae, Fagaceae, Bignoniaceae, and Sapindaceae of Angiospermae and most of the families of the Gymnospermae produces FAs with more than 18 carbon atoms (Smith, 1971; Takagi & Itabashi, 1982; Voelker & Kinney, 2001). Gymnospermae members are also known for their unusual double bond positions (Takagi & Itabashi, 1982). Although rare in plants, members of Poaceae (*Lolium perenne*), Sterculiaceae (*Sterculia foetida*, *Sterculia alata*), Bignoniaceae (*Cuspidaria pterocarpa*), Malvaceae (*Hybiscus syriacus*), Santalaceae (*Acanthosyris spinescens*), and Sapindaceae (*Euphoria longans*) are reported to contain odd number of carbon atoms (C13 to C31) in the FAs (Body & Hansen, 1978; Diedrich & Henschel, 1990; Smith, 1971). Due to the specific nature of uFAs, they are associated with functional relevance like toxicity or indigestibility to protect the seeds from herbivory and is generally found in the seeds of the families Apiaceae, Araliaceae and Garryaceae (van de Loo et al., 2018).

## 5 NONEDIBILITY FACTORS IN OILSEED PLANTS

Castor (*Ricinus communis*) is one of the promising nonedible oilseed crops with nearly 50% oil content by weight of the seed (Mubofu, 2016), and with a potential yield of 2.0 tons per hectare (NMOOP, 2018). Presence of toxic components like ricin, hemagglutinin, Ric C1 and C3, and ricinine are reported in castor (Liu et al., 2021; Lord et al., 1994; Waller et al., 1965; Youle & Huang, 1978). Similarly, tree‐of‐paradise or aceituno (*Simarouba glauca*) is another promising source for oil with 60–70% oil content (Govindaraju et al., 2009; Rout et al., 2014) and with a potential yield of around 5.0 tons per hectare (Joshi & Hiremath, 2000). Although the seeds contain quassinoids (Govindaraju et al., 2009; Monseur & Motte, 1983; Osagie‐Eweka et al., 2021), a toxic compound of the triterpene family, the oil is reported to be free of toxic compounds and is edible (Lewy‐van Séveren, 1953). However, the seed meal would still contain the toxic compound after oil extraction and needs detoxification treatments for secondary uses.

## 6 OMIC TOOLS TO GENERATE NEWER ALTERNATIVE SOURCES FOR EDIBLE OIL

There are more than 20 species available as the sources for edible oilseeds (Table 1). However, oil from only seven species (soybean, rapeseed, cotton, sunflower, peanut, oil palm, and copra) contributes to more than 95% of the world production and market (Vinnichek et al., 2019). The major reasons for only seven of the 20 species being used as a source of edible oil are one or more of the following: Lesser oil content, shorter shelf life, not widely accepted across different regions though edible like drumstick (Tsaknis et al., 1999) and aceituno (Lewy‐van Séveren, 1953). Consequently, enriching and diversifying the seed oil sources with wider consumer acceptability would potentially help meet the growing oil demand. Species with more than 50% seed oil content and fruit yields above one ton per hectare would be the most promising newer alternative sources for edible oil (Table 1, *Callophyllum*, *Madhuca*, *Pongamia*, *Azadirachta*) if we could succeed in modifying the desired plant using genetic engineering technologies. Reports indicate the possibility of overcoming the post‐translational regulation issues through using a set of key TAG biosynthetic genes than a single desired gene (Bates et al., 2014). However, for those species that produce toxins or other undesirable compounds (Table 1, *Ricinus*, *Simarouba*, *Pongamia*, *Azadirachta*), different set of strategies are required.

**TABLE 1:** Oil content and fatty acid composition for non‐edible and edible oilseed crops

Species name	Family	Fatty acid composition (wt. %)						Oil content (%w)	References
		Saturated	Unsaturated						
			18:1	18:2	18:3	Other	Total		
**Nonedible oil crops**									
*Callophyllum inophyllum*	Calophyllaceae	**24.9**	34.1	38.3	0.3		**72.7**	65	(Islam et al., 2018)
*Jatropha curcas*	Euphorbiaceae	**22.5**	43.5	34.4	0.8		**78.7**	55	(Islam et al., 2018)
*Madhuca indica*	Sapotaceae	**31.8**	46.3	17.9			**64.2**	50	(Islam et al., 2018)
*Ricinus communis*	Euphorbiaceae	**2**	3	4.2	0.3	90^a^	**97.5**	50	(Mubofu, 2016)
*Azadirachta indica*	Meliaceae	**29.3**	61.9	7.5			**69.4**	44.5	(Islam et al., 2018)
*Simmondsia chinesis*	Simmondsiaceae	**24.4**	54.2	1.6			**55.8**	39–45	(Arya et al., 2016)
*Pongamia pinnata*	Fabaceae	**19.2**	51.6	16.5	2.7		**70.8**	33	(Islam et al., 2018)
*Sapindus mukorossi*	Sapindaceae	**6.12**	52.64	4.73	1.94		**59.31**	30	(Chhetri et al., 2008)
*Cyperus esculentus*	Cyperaceae	**17.55**	69.32	13.11	0		**82.43**	20–36	(Zhang et al., 1996)
**Edible oil crops**									
*Cocos nucifera*	Arecaceae	**81.2–94**	5.0–10	1–2.5	0.2–2.5		**6.2–15**	65–74	(Marina et al., 2009; Xiao et al., 2019)
*Elaeis guineensis*	Arecaceae	**86.97**	11.28	1.55			**12.83**	65–70	(Tambun et al., 2019)
*Simarouba glauca*	Simaroubaceae	**40.8–42.3**	54.6	2.3	0.2		**57.1**	60–70	(Rout et al., 2014)
*Juglans regia*	Juglandaceae	**5.0–17**	10.0–20	55–70	10.0–18.0		**75–95**	50–70	(Hayes et al., 2016; Özcan et al., 2010; Sena‐Moreno et al., 2016)
*Elaeis guineensis*	Arecaceae	**49.7–57.5**	37.3–40.8	9.1–11.0	0.01–0.25		**46.41–50.3**	50–55	(Kasemsumran et al., 2012; Nehdi et al., 2018)
*Arachis hypogaea*	Fabaceae	**9.9–13.8**	37.0–55.6	25.3–39.7	0.40–3.2		**62.7–91.1**	46–57	(Sui et al., 2018; Wang et al., 2018)
*Helianthus annuus*	Asteraceae	**9.0–13**	16.4–27.6	60.2–72.1	0.07–1.8		**76.67–91**	46–50	(Orsavova et al., 2015; Rauf et al., 2017)
*Brassica rapa*	Brassicaceae	**4**	16	15	9	53#	**93**	47.3	(Cartea et al., 2019; Velasco et al., 1998)
*Guizotia abyssinica*	Asteraceae	**16.57**	6.06	76.43	0.22		**82.71**	42–44%	(Deme et al., 2021; Mekonnen et al., 2018)
*Brassica napus*	Brassicaceae	**6**	15	14	9	52#	**90**	42.5	(Cartea et al., 2019; Dyer et al., 2008)
*Sesamum indicum*	Pedaliaceae	**12.4–14.4**	36.7–42.94	43.2–54.0	0.2–0.95		**80.1–87.6**	43–61	(Ghosh et al., 2019; Hashempour‐Baltork et al., 2018; Latif & Anwar, 2011)
*Moringa oleifera*	Moringaceae	**21.18**	76.73	0.76	0.46		**71.70–80.70**	36.7	(Leone et al., 2016; Tsaknis et al., 1999)
*Camellia hainanica*	Theaceae	**7.7–12.9**	74.3–83.6	7.0–15	0.2–0.4		**81.5–92.3**	37.60–41.60	(Lin et al., 2018; Wang et al., 2011; Zhenggang et al., 2021)
*Linum usitatissimum*	Linaceae	**8**	19	24	47		**90**	35–45	(Dyer et al., 2008; Tavarini et al., 2019)
*Olea europaea*	Oleaceae	**12.5–20.9**	54.5–80.2	4.9–21.2	0.7–1.5		**60.1–87.5**	31–56	(Geng et al., 2018; Olmo‐García et al., 2018; Sun et al., 2017)
*Paeonia officinalis*	Paeoniaceae	**6.2–12.4**	20.5–45.1	16.5–33.6	28.1–46.9		**65.1–93.8**	27–33	(Llorent‐Martínez et al., 2011; Ning et al., 2015; Wei et al., 2018; Zhang et al., 2019)
*Carthamus tinctorius*	Asteraceae	**5**	8	87			**95**	23–36	(Griffiths et al., 1988; Ichihara & Noda, 1980; Khalid et al., 2017)
*Glycine max*	Fabaceae	**6.0–24.0**	15–36	42.8–56.1	2.0–14		**59.8–94**	18.0–24	(Li et al., 2018; Wijewardana et al., 2019)
*Gossypium hirsutum*	Malvaceae	**27.5–33.7**	16.5–27.0	43.2–54.0	0.13–0.3		**59.83–72.5**	15–40	(Shang et al., 2017; Talpur et al., 2014; Wang et al., 2016)
*Perilla frutescens*	Lamiaceae	**7.2–5.9**	9.3–20	10.0–24	47–64		**66.3–92.8**	17–42.7	(Kim et al., 2019)
*Persea americana*	Lauraceae	**28.9**	50.95	13.87	0.58		**65.1**	11–18.80	(Gómez‐López, 2002; Ranade & Thiagarajan, 2015)
*Zea mays*	Poaceae	**15.0–16**	27.6–34.6	48.6–55.3	0.60–1.49		**76.8–85**	4.5–4.8	(Wang et al., 2010; Weinstock et al., 2006)

^a^ 18:1OH (Ricinoleic acid), #20:1 + 22:1(eicosenoic acid + docosenoic acid).

*Note*: The bold values indicate the total saturated and unsaturated FA composition. The unsaturated bold values are the sum of the values of the four columns (in a row), 18:1, 18:2, 18:3, and others.

Although a few of the toxins present in the seeds do not get extracted along with the oil during the extraction process, they will still be present in the seed meal in higher concentrations (30–60% depending on the oil content). This requires scientific interventions to develop methods that would neutralize or detoxify the toxic components for further utilization. To overcome, the available and affordable breeding or biotechnological tools could potentially be used to develop plants that are free from toxicity (Auld et al., 2001; Sujatha et al., 2008). For this, the first step is to understand the biosynthesis and key regulatory checkpoints of the toxic components, in addition to the TAG biosynthesis. Transcriptional repression or activation for these regulatory check points or enhancing the transcriptional levels of the genes of the oil biosynthesis would lead to enhanced oil production. Wrinkled1 (WRI1) is one of the key transcriptional factors regulating FA biosynthesis in plants (Table S1) (Kong et al., 2020). In oilseed plants, WRI1 is the most studied TF, followed by Leafy cotyledon (LEC), Abscisic acid insensitive (ABI), and bZIP TFs documented to regulate FA biosynthesis (Table S1). Also, RNAi or CRISPR‐mediated gen(om)e editing tools to block the pathway of toxin production would yield promising results.

## 7 CONCLUSION AND FUTURE PERSPECTIVES

With the advent of next‐generation sequencing technology and associated development in the computational tools, the cost of sequencing has become significantly less and affordable to generate a high quality genome sequence (Guo, 2021). With reference to seed oil producing plants, most of the species are sequenced at the whole genome level or, at the least, at the transcriptome level, to understand the oil biosynthetic pathways (Table S1). Despite being the primary metabolic pathway, TAG biosynthesis is regulated at multiple levels with multiple checkpoints; starting from ACCase to the LD biogenesis, the final step is well known (Bates et al., 2014; Chapman et al., 2019; Chen et al., 2021; Li et al., 2017; Lu et al., 2009, 2021; Weselake et al., 2009; Zhai et al., 2021). On the other hand, the biosynthetic pathway for the toxins or the components in the seed or the oil that makes them not preferred by consumers is comparatively less understood and requires more attention and research. Understanding the toxins' or the nonpreferred metabolites' pathways will help modulate plants that are free of toxins or nonpreferred metabolites and more oilseed species of nonedible type could be converted to edible type. Most of these nonedible oilseed species have potential yields much higher than the commonly cultivated oilseed crops with yields less than a ton per hectare. However, when the nonedible oilseed species are suitably converted, this would give a higher oil yield (and edible too) and would fetch larger returns. This will help diversify the plant sources of oil for eating and other industrial purposes.

Of the three TAG biosynthesis pathways known in plants, de novo biosynthesis or the Kennedy pathway, PC‐derived biosynthesis, and cytosolic biosynthesis or the Rajasekharan pathway the first two pathways exhibit variability in the pathway between phosphatidic acid and TAG only, causing modified FA in the DAG and thereby leading to the diversity in oil composition while the LD biogenesis is common for both the pathways. Here, except the PC‐derived biosynthesis, which is acyl‐CoA‐independent, others are acyl‐CoA‐dependent. In the case of cytosolic biosynthesis, the pathway leading to TAG formation is different and occurs in the cytosol. Not much is known about LD biogenesis for the TAG molecules formed through the cytosolic pathway. How it is mobilized into the phospholipid monolayer and the assembly of LD with its associated proteins (LDAP) in the cytosol, for the cytosol derived TAG, require scientific insights.

Understanding the gene regulatory networks in plants that produce oil in the endosperm (Reynolds et al., 2019) might help define strategies to synthesize oil in the endosperm of the cereal crops and other crops where the endosperm is chlorophyllous in nature (Rangan, 2020, and citations thereof). Utilizing the strengths of endosperm‐specific callus cell cultures could be a commercially viable option in the near future with successful demonstration of developing cell cultures producing TAG (Carmona‐Rojas et al., 2021).

## CONFLICT OF INTEREST

The authors declare no conflict of interest associated with the work described in this manuscript.

## Supporting information


**Table S1.** Genes and transcription factors of TAG biosynthesis identified in plant species.
