# Supplementary material for: Can omic tools help generate alternative newer sources of edible seed oil?
Source: Plant Direct. 2022 Jun 7;6(6):e399. doi: 10.1002/pld3.399 (PMC9219012; doi:10.1002/pld3.399)
Supplement: Supplementary file 1 — Table S1. Genes and transcription factors of TAG biosynthesis identified in plant species. [file PLD3-6-e399-s001.docx]

**Table S1.** Genes and transcription factors of TAG biosynthesis identified in plant species.

| **S.No.** | **Common name** | **Genes** | **Transcription Factors** | **References** |
| --- | --- | --- | --- | --- |
| 1. | Cotton (+, *) | *Beta-KASII*, *SAD*, *PEPC1*, *GPAT*, *PDAT*, *FAD2-1 and FATB*, *CIPKs*, *LPAAT.* | WRI1, NF-YB6, DPBF2. | (Liu, *et al*., 2017)  (Liu, *et al*., 2019)  (Xu, *et al*., 2016)  (Shockey, *et al*., 2016)  (Zang, *et al*., 2019)  (Liu, *et al*., 2017)  (Cui, *et al*., 2020)  (Wang, *et al*., 2017)  (Zhu, *et al*., 2021) |
| 2. | Peanut (+, *) | *LEC1*, *ACCase A (ACC II)*, *ACCase (BC4)*, *KAS II*, *SAD*, *DGAT*, *Ole*, *GPAT9 gene.* | LEC1, LEC2, WRI 1, bZIP | (Tang, *et al*., 2018)  (Lv, *et al*., 2020) |
| 3. | Sunflower (+, *) | *KCSs*, *Oleosins.* | WRI1 | (González-Mellado, *et al*., 2019)  (Moreno-Pérez, *et al*., 2021) |
| 4. | Soybean (+, *) | *ABI3b*, *NFYA*, *FAD2-1B*, *DGAT1*, *SWEET39*, *Oleo1*, *FATB1a*, *PDAT*, *PLDα1*, *GPAT* family genes, *DGAT2B*, *SDP1.* | LEC2 | (Yang, *et al*., 2019)  (Zhang, *et al*., 2018)  (Miao, *et al*., 2020)  (Zhang, *et al*., 2019)  (Liu, *et al*., 2020)  (Eskandari, *et al*., 2013)  (Kanai, *et al*., 2019)  (Manan, *et al*., 2017) |
| 5. | Rapeseed (+, *) | *ACCase*, *FatA*, *FatB*, *FAD2*, *FAD3*, *DGAT*, *SFAR gene*, *Acyl-CoA synthetase2*, *GPDH*, *GPAT*, *ScLPAAT.* | BnWRI1, BnLEC1, BnLACS2, AtGDSL1, BnGDSL1, WRKY6 | (Sharma, *et al*., 2012)  (Karunarathna, *et al*., 2020)  (Ding, *et al*.,2020)  (Liu, *et al*.,2015)  (Wu, *et al*., 2014)  (Ding, *et al*., 2019)  (Song, *et al*., 2020) |
| 6. | Hemp (+, *) | *Delta 12 and delta 15 desaturase genes.* |  | (Bielecka, *et al*., 2014) |
| 7. | Corn (+, *) | *OBAP1*, *DGAT*, *SAD1 gene*. | WRI1, TCP4, LEC1 | (López-Ribera, *et al*., 2014)  (Yan, *et al*., 2018)  (Du, *et al*., 2016)  (Shen, *et al*., 2010) |
| 8. | Sesame (+, *) | *GPAT and DGAT genes*, *ACCase*,*ACP*,*PDAT*, and *PDCT*, *LPAAT*, *NsLTPs*, *caleosin*, *steroleosin and Oleosins.* |  | (Wang, *et al*., 2019)  (Lin, *et al*., 2005)  (Song, *et al*., 2021) |
| 9. | Rice (+, *) | *DGAT1.* | WRI1, | (Bhunia, *et al*., 2021) |
| 10. | Perilla (*) | *BCCP α- CT*, *BCβ-CT*, *MCAAT*, *KASIII*, *KASI*, *KAR*, *HAD*, *EAR, FATB, FATA*, *AAD*, *PCH*, *G3PDH*, *GPAT*, *LPAAT*, *PAP*, *DGAT*, *PDCT*, *PDAT*, *FAD2*, *LPCAT.* | WRI1, LEC1, FUS3 ABI3and GL2. | (Liao, *et al*., 2018) |
| 11. | Fiber flax (*) | *LPAAT*, *β-ketoacyl CoA synthase*, *FAE*, *SAD and FAD*, *OLEOSIN.* |  | ([Hall](https://www.sciencedirect.com/science/article/pii/B9781893997981000063#!) *et al*., 2016) |
| 12. | Safflower (*) | *DHLAT*, *α-PDHC*, *β-PDHC*, *PDP*, *α-CT*, *β-CT*, *BCCP*, *MCAAT*, *FAD6*, *FAD7/8*, *Oleate desaturase*, *LACS*, *LPCAT /lyso-PAF acetyltransferase*, *PDAT*, *Diacylglycerol choline phosphotransferase.* |  | (Li, *et al*., 2021) |
| 13. | Olive (+, *) | *bZIPs*, *LPG genes*, *KASII.6 KASIII.1*, *LPAAT2*, *DGAT1*, *ACC.1*, *BCCP.1*, *SACPD 1*, 2,*4*, *FAD2 4,5*, *GPAT5.* | bZIP | (Rong, *et al*., 2020) |
| 14. | Tea- oil tree (*) | *Mcs*, *cmk*, *hdr*, *hds*, *dxs*, *dxr*, *ggpps*, *and gpps*; *hmgr*, *fpps*, *mvk and pmd*; *mTPS*, *families TPSb*, *TPSg*; *sTPS, gene family TPSa*, *dxs2*, *mct*, *hmgs1 and ippi1 and ippi2.* | MYB-like, AP2/ERF, MYC and WRKY classes. | (Webb, *et al*., 2013) |
| 15. | Oil palm (+, *) | *PDH*, *ACCase*, *MA*, *KAS*, *ACP*, *KAR*, *HAD*, *EAR*, *TECR*, *SAD*, *FAT*, *G3PDH*, *LACS*, *GPAT*, *LPAAT*, *PAP*, *DGAT*, *CPT*, *PDCT*, *PC: DAG*, *PDAT*, *LPCAT*, *FAE1*, *FAD.* | EgWRI1-1, EgNF-YA3, EgNF-YC2 and EgABI5. | (Yeap, *et al*., 2017) |
| 16. | Coconut oil (+, *) | *FAS*, *FATA*, *FATB*, *GPAT*, *LPAAT*, *DGAT*, *PDAT*, *PC*, *DAG*. | CoWRI1, an AP2/ EREBP. | (Reynolds, *et al*., 2019)  (Sun, *et al*., 2017) |
| 17. | Peony (*) | *GPAT*, *LPAAT*, *PAP*, *DGAT*, *LPCAT*, *PlcC*, *PLA2*, *ω-6 FADs*, *ω-3 FADs*, *ACCase*, *Stearoyl-ACP desaturase gene (PoSAD)*, *OLEs*. |  | (Xiu, *et al*., 2018) |
| 18. | Pecan (+, *) | *ACCase*, *MAT*, *KASIII*, *KASI*, *KAR*, *HAD*, *EAR*, *FATA*, *FATB*, *SAD*, *GPAT*, *LPAAT*, *DGAT*, *PDCT*, *LACS*, *FAD* family genes*.* | WRI1*and*bZIP67 | (Zhang, *et al*., 2021) |
| 19. | Walnut (+, *) | *ACCase*, *KASIII*, *KASI*, *KAR*, *FATA*, *FATB*, *SAD*, *ACOT*, *PAS2*, *KCR*, *ECR*, *DGAT*, *PDAT*. |  | (Zhao, *et al*., 2020) |
| 20. | Idesia (*) | *ACC*, *GPAT*, *LPAT*, *PAP*, *DGAT*, *PDAT*, *SSI2*, *FAD4*, *FAD5*, *FAD6*, *FAD7*, *FAD8*, *FAD2 and FAD3*, *FATA and FATB*, *LACS*. | WRI1*,* LEC1, LEC2, ABI4, ABI3, and FUS3. | (Li, *et al*., 2016) |
| 21. | Cocoa (+, *) | *ACC*, *ACP*, *MAT*, *FAS*, *SAD*, *GPAT*, *DGAT*, *LPAT*, *PAP*, *PDAT*, *GPDH.* |  | (Li *et al*., 2019) |
| 22. | Ratanjyot (+, *) | *Oleo 1*, *Oleo 2*, *PDAT*, *DGK1*, *ECH*, *KCR2*, *KCS*, *Lipase*, *SD*, *CLK*  *LACS8*. |  | (Xu *et al*., 2011), (Gu *et al*., 2012), (Sood *et al*., 2015) |
| 23. | Jojoba (*) | *MCAL*, *ACCase- α*, *BC*, *BCCP*, *ACCase- β*, *MCAAT*, *KAS I*, *KAS II*, *KAR4*, *HAD*, *EAR*, *AAT(FATB)*, *LACS*, *FAD2*, *FAD6*, *SLD*, *STE*, *FAD4*, *FAD5*, *FAD6*, *SAD6*, *KCS*, *KCR*, *HCD*, *ECR*, *FAR*, *FAO*, *G3PDH*, *GPAT*, *LPAAT*. |  | (Alotaibi *et al*., 2020) |
| 24. | Karanja (*) | *ACC-CTα*, *ACC-CTβ*, *ACC-BC*, *ACC-BCCP*, *ACC1*, *MAT*, *KASIII*, *KAR*, *HAD*, *EAR*, *KASI*, *KASII*, *SAD*, *FATA*, *FATB*, *LACS*, *GPAT*, *LPAT*, *PAH1*, *PAH2*, *DGAT1*, *DGAT2*, *DGAT3*, *PDAT*, *PDCT*, *FAD2*, *FAD3*, *FAD6*, *FAD7*, *SDP1*, *ACD*, *ECH*, *HDH*, *KAT*, *Ole.* | WRI1, FUS3, ABI4*.* | (Sreeharsha *et al*.*,* 2016; Huang *et al*., 2018) |
| 25. | Castor (+, *) | *FAH*, *RcACS2*, *RcFATB*,  *RcDGAT1*. | RcWRI1A, RcWRI1 B | (Ji *et al*., 2018; Van De Loo, *et al*., 1995; Wan, *et al*., 2019) |

**+:** indicates availability of whole genome sequence; ***:** indicates availability of transcriptomic sequence

**Abbreviations**

**ABI3:** Abscisic acid-insensitive 3; **ACCase:** Acetyl CoA carboxylase; **ACOT:**acyl-CoA thioesterase; **ACP:** Acyl carrier proteins; **BCCP:** Biotin carboxyl carrier protein; **bZIP:** Basic leucine zipper (bZIP) gene family; **CIPKs:** Calcineurin B- like protein- interacting protein kinases; **CLK:** Choline kinase; **CT:** Carboxyl transferase; **DGAT:** Diacylglycerol acyltransferases; **DGK:** Diacylglycerol kinase; **ECH:** Enoyl-CoA hydratase; **ECR:** very-long-chain enoyl-CoA reductase; **FATB:** Fatty acid thioesterases B; **FAD:** Fatty acid desaturase; **FAD2-1:** Fatty acid desaturase-2-1; **FAE:** Fatty acid elongase; **FAO:** long-chain-alcohol oxidase FAO1; **FAR:** alcohol-forming fatty acyl-CoA reductase; **FAS:** fatty acid synthase; **GPAT:** Glycerol-3- Phosphate Acyltransferase; **GPDH:** glycerol-3-phosphate; dehydrogenase; **HAD:** hydroxyacyl-ACP dehydrase; **HCD:** very-long-chain (3R)-3-hydroxyacyl-CoA dehydratase; **HDH:** 3-hydroxyacyl-CoA dehydrogenase; **KAR:** ketoacyl-ACP reductase; **KASII:** Ketoacyl-ACP synthase II; **KCR:** 3-ketoacyl-CoA reductase isoform 2; **KCSs:** Ketoacyl CoA synthases gene; **LACS:** long-chain acyl-CoA synthetase; **LEC1:** LEAFY COTYLEDON 1; **LPAAT:** Lysophosphatidate acyltransferase gene; **LPCAT:** Lysophosphatidylcholine acyltransferase; **MAT:** malonyl-CoA: acyl carrier protein S-malonytransferase; **MCAL:** malonate-CoA ligase; **MCMT:**malonyl-CoA ACP trans acylase; **NFYA:** Nuclear Factor-Y Subunit A; **nsLTPs:** nonspecific lipid-transfer proteins; **OBAP1:** oil body associated protein1; **OLEO1:** Oleosin protein encoding gene; **PAP:** Phosphatidic acid phosphatase; **PAS2:**very long-chain (3R)-3-hydroxyacyl-CoA dehydratase 2; **PDAT:** Phospholipid: Diacylglycerol acyltransferase gene; **PDCT:** phosphatidylcholine: diacylglycerol choline phosphotransferase; **PDH:** pyruvate dehydrogenase; **PEPC1:** Phosphoenolpyruvate Carboxylase 1; **PLA2:** phospholipase A2; **SAD:** Stearoyl- acyl carrier protein desaturase gene; **SD:** Sterol desaturase; **SDP1:** SUGAR DEPENDENT1; **SFAR:** SEED FATTY ACID REDUCER genes; **SLD:** delta (8)-fatty-acid desaturase 2; **STE:** delta(7)-sterol-C5(6)-desaturase; **TECR:** trans-2,3-enoyl-CoA reductase; **WRI 1:** WRINKLED1.

**References:**

**Alotaibi, S.S., Elseehy, M.M., Aljuaid, B.S. and El-Shehawi, A.M**. 2020. Transcriptome analysis of jojoba (*Simmondsia chinensis*) during seed development and liquid wax ester biosynthesis. *Plants*, ***9*(5)**, p.588.

**Bhunia, R.K., Sinha, K., Chawla, K., Randhawa, V. and Sharma, T.R**. 2021. Functional characterization of two type-1 diacylglycerol acyltransferase (DGAT1) genes from rice (*Oryza sativa*) embryo restoring the triacylglycerol accumulation in yeast. *Plant Molecular Biology*, ***105*(3)**, pp.247-262.

**Bielecka, M., Kaminski, F., Adams, I., Poulson, H., Sloan, R., Li, Y., Larson, T.R., Winzer, T. and Graham, I.A**. 2014. Targeted mutation of Δ12 and Δ15 desaturase genes in hemp produce major alterations in seed fatty acid composition including a high oleic hemp oil. *Plant Biotechnology Journal*, ***12*(5)**, pp.613-623.

**Cui, Y., Su, Y., Wang, J., Jia, B., Wu, M., Pei, W., Zhang, J. and Yu, J**. 2020. Genome-wide characterization and analysis of CIPK gene family in two cultivated allopolyploid cotton species: sequence variation, association with seed oil content, and the role of GhCIPK6. *International Journal of Molecular Sciences*, ***21*(3)**, p.863.

**Ding, L.N., Gu, S.L., Zhu, F.G., Ma, Z.Y., Li, J., Li, M., Wang, Z. and Tan, X.L**. 2020. Long-chain acyl-CoA synthetase 2 is involved in seed oil production in *Brassica napus*. *BMC Plant Biology*, ***20*(1)**, pp.1-14.

**Ding, L.N., Guo, X.J., Li, M., Fu, Z.L., Yan, S.Z., Zhu, K.M., Wang, Z. and Tan, X.L**. 2019. Improving seed germination and oil contents by regulating the GDSL transcriptional level in *Brassica napus*. *Plant Cell Reports*, ***38*(2)**, pp.243-253.

**Du, H., Huang, M., Hu, J. and Li, J**. 2016. Modification of the fatty acid composition in *Arabidopsis* and maize seeds using a stearoyl-acyl carrier protein desaturase-1 (ZmSAD1) gene. *BMC Plant Biology*, ***16*(1)**, pp.1-10.

**Eskandari, M., Cober, E.R. and Rajcan, I**. 2013. Using the candidate gene approach for detecting genes underlying seed oil concentration and yield in soybean. *Theoretical and Applied Genetics*, ***126*(7)**, pp.1839-1850.

**González-Mellado, D., Salas, J.J., Venegas-Calerón, M., Moreno-Pérez, A.J., Garcés, R. and Martínez-Force, E**. 2019. Functional characterization and structural modelling of *Helianthus annuus* (sunflower) ketoacyl-CoA synthases and their role in seed oil composition. *Planta*, ***249*(6)**, pp.1823-1836.

**Gu, K., Yi, C., Tian, D., Sangha, J.S., Hong, Y. and Yin, Z**. 2012. Expression of fatty acid and lipid biosynthetic genes in developing endosperm of *Jatropha curcas*. *Biotechnology for Biofuels*, ***5*(1)**, pp.1-15.

**Hall, L.M., Booker, H., Siloto, R.M., Jhala, A.J. and Weselake, R.J**. 2016. Flax (*Linum usitatissimum* L.). In *Industrial Oil Crops* (pp. **157-194**). AOCS Press.

**Huang, J., Hao, X., Jin, Y., Guo, X., Shao, Q., Kumar, K.S., Ahlawat, Y.K., Harry, D.E., Joshi, C.P. and Zheng, Y**. 2018. Temporal transcriptome profiling of developing seeds reveals a concerted gene regulation in relation to oil accumulation in *Pongamia* (*Millettia pinnata*). *BMC Plant Biology*, ***18*(1)**, pp.1-16.

**Ji, X.J., Mao, X., Hao, Q.T., Liu, B.L., Xue, J.A. and Li, R.Z**. 2018. Splice variants of the castor WRI1 gene upregulate fatty acid and oil biosynthesis when expressed in tobacco leaves. *International Journal of Molecular Sciences*, ***19*(1)**, p.146.

**Kanai, M., Yamada, T., Hayashi, M., Mano, S. and Nishimura, M**. 2019. Soybean (Glycine max L.) triacylglycerol lipase GmSDP1 regulates the quality and quantity of seed oil. *Scientific Reports*, ***9*(1)**, pp.1-10.

**Karunarathna, N.L., Wang, H., Harloff, H.J., Jiang, L. and Jung, C**. 2020. Elevating seed oil content in a polyploid crop by induced mutations in SEED FATTY ACID REDUCER genes. *Plant Biotechnology Journal*, ***18*(11)**, pp.2251-2266.

**Li D, Wang Q, Xu X, Yu J, Chen Z, Wei B, Wu W**. 2021. Temporal transcriptome profiling of developing seeds reveals candidate genes involved in oil accumulation in safflower (*Carthamus tinctorius* L.). *BMC Plant Biol*. **21(1)**:181.

**Li, F., Wu, B., Yan, L., Hao, C., Qin, X., Lai, J. and Song, Y**. 2019. Transcriptional profiling reveals differentially expressed genes involved in lipid biosynthesis during cacao seed development. *Scientific Reports*, ***9*(1)**, pp.1-11.

**Li, R.J., Gao, X., Li, L.M., Liu, X.L., Wang, Z.Y. and Lü, S.Y**. 2016. De novo assembly and characterization of the fruit transcriptome of *Idesia polycarpa* reveals candidate genes for lipid biosynthesis. *Frontiers in Plant Science*, ***7***, p.801.

**Liao B, Hao Y, Lu J, Bai H, Guan L, Zhang T**. 2018. Transcriptomic analysis of *Perilla frutescens* seed to insight into the biosynthesis and metabolic of unsaturated fatty acids. *BMC Genomics*. **19(1)**:213.

**Lin, L.J., Liao, P.C., Yang, H.H. and Tzen, J.T**. 2005. Determination and analyses of the N-termini of oil-body proteins, steroleosin, caleosin and oleosin. *Plant Physiology and Biochemistry*, ***43*(8)**, pp.770-776.

**Liu, B., Sun, Y., Xue, J., Mao, X., Jia, X. and Li, R**. 2019. Stearoyl-ACP Δ9 desaturase 6 and 8 (GhA-SAD6 and GhD-SAD8) are responsible for biosynthesis of palmitoleic acid specifically in developing endosperm of upland cotton seeds. *Frontiers in Plant Science*, ***10***, p.703.

**Liu, F., Xia, Y., Wu, L., Fu, D., Hayward, A., Luo, J., Yan, X., Xiong, X., Fu, P., Wu, G. and Lu, C**. 2015. Enhanced seed oil content by overexpressing genes related to triacylglyceride synthesis. *Gene*, ***557*(2)**, pp.163-171.

**Liu, F., Zhao, Y.P., Zhu, H.G., Zhu, Q.H. and Sun, J**. 2017. Simultaneous silencing of GhFAD2-1 and GhFATB enhances the quality of cottonseed oil with high oleic acid. *Journal of Plant Physiology*, ***215***, pp.132-139.

**Liu, J.Y., Li, P., Zhang, Y.W., Zuo, J.F., Li, G., Han, X., Dunwell, J.M. and Zhang, Y.M**. 2020. Three‐dimensional genetic networks among seed oil‐related traits, metabolites and genes reveal the genetic foundations of oil synthesis in soybean. *The Plant Journal*, ***103*(3)**, pp.1103-1124.

**López-Ribera, I., La Paz, J.L., Repiso, C., García, N., Miquel, M., Hernández, M.L., Martínez-Rivas, J.M. and Vicient, C.M**. 2014. The evolutionary conserved oil body associated protein OBAP1 participates in the regulation of oil body size. *Plant Physiology*, ***164*(3)**, pp.1237-1249.

**Lv Y, Zhang X, Luo L, Yang H, Li P, Zhang K, Liu F, Wan Y**. 2020.Characterization of glycerol-3-phosphate acyltransferase 9 (AhGPAT9) genes, their allelic polymorphism and association with oil content in peanut (*Arachis hypogaea* L.). *Scientific Reports*; **10(1)**:14648.

**Manan, S., Ahmad, M.Z., Zhang, G., Chen, B., Haq, B.U., Yang, J. and Zhao, J**. 2017. Soybean LEC2 regulates subsets of genes involved in controlling the biosynthesis and catabolism of seed storage substances and seed development. *Frontiers in Plant Science*, ***8***, p.1604.

**Miao, L., Yang, S., Zhang, K., He, J., Wu, C., Ren, Y., Gai, J. and Li, Y**. 2020. Natural variation and selection in GmSWEET39 affect soybean seed oil content. *New Phytologist*, ***225*(4)**, pp.1651-1666.

**Moreno-Pérez, A.J., Martins-Noguerol, R., DeAndrés-Gil, C., Venegas-Calerón, M., Sánchez, R., Garcés, R., Salas, J.J., Troncoso-Ponce, M.A., Tena, J.J., Santos-Pereira, J.M. and Martínez-Force, E**. 2021. Genome-Wide Mapping of Histone H3 Lysine 4 Trimethylation (H3K4me3) and Its Involvement in Fatty Acid Biosynthesis in Sunflower Developing Seeds. *Plants*, ***10*(4)**, p.706.

**Reynolds, K.B., Cullerne, D.P., El Tahchy, A., Rolland, V., Blanchard, C.L., Wood, C.C., Singh, S.P. and Petrie, J.R**. 2019. Identification of genes involved in lipid biosynthesis through de novo transcriptome assembly from *Cocos nucifera* developing endosperm. *Plant and Cell Physiology*, ***60*(5)**, pp.945-960.

**Rong, S., Wu, Z., Cheng, Z., Zhang, S., Liu, H. and Huang, Q**. 2020. Genome-wide identification, evolutionary patterns, and expression analysis of bZIP gene family in olive (*Olea europaea* L.). *Genes*, ***11*(5)**, p.510.

**Sharma, A. and Chauhan, R.S**. 2012. In silico identification and comparative genomics of candidate genes involved in biosynthesis and accumulation of seed oil in plants. *Comparative and Functional Genomics*, **2012**: 914843 (doi: 10.1155/2012/914843).

**Shen, B., Allen, W.B., Zheng, P., Li, C., Glassman, K., Ranch, J., Nubel, D. and Tarczynski, M.C**. 2010. Expression of ZmLEC1 and ZmWRI1 increases seed oil production in maize. *Plant Physiology*, ***153*(3)**, pp.980-987.

**Shockey, J., Regmi, A., Cotton, K., Adhikari, N., Browse, J. and Bates, P.D**. 2016. Identification of *Arabidopsis* GPAT9 (At5g60620) as an essential gene involved in triacylglycerol biosynthesis. *Plant Physiology*, ***170*(1)**, pp.163-179.

**Song, G., Li, X., Munir, R., Khan, A.R., Azhar, W., Yasin, M.U., Jiang, Q., Bancroft, I. and Gan, Y**. 2020. The WRKY6 transcription factor affects seed oil accumulation and alters fatty acid compositions in *Arabidopsis thaliana*. *Physiologia Plantarum*, ***169*(4)**, pp.612-624.

**Song, S., You, J., Shi, L., Sheng, C., Zhou, W., Dossou, S.S.K., Dossa, K., Wang, L. and Zhang, X**. 2021. Genome-Wide Analysis of nsLTP Gene Family and Identification of SiLTPs Contributing to High Oil Accumulation in Sesame (*Sesamum indicum* L.). *International Journal of Molecular Sciences*, ***22*(10)**, p.5291.

**Sood A,Chauhan RS**. 2015. Regulation of FA and TAG biosynthesis pathway genes in endosperms and embryos of high and low oil content genotypes of *Jatropha curcas* L. *Plant Physiol Biochem*. **94**:253-67.

**Sreeharsha, R.V., Mudalkar, S., Singha, K.T. and Reddy, A.R**. 2016. Unravelling molecular mechanisms from floral initiation to lipid biosynthesis in a promising biofuel tree species, *Pongamia pinnata* using transcriptome analysis. *Scientific Reports*, ***6*(1)**, pp.1-15.

**Sun, R., Ye, R., Gao, L., Zhang, L., Wang, R., Mao, T., Zheng, Y., Li, D. and Lin, Y**. 2017. Characterization and ectopic expression of CoWRI1, an AP2/EREBP domain-containing transcription factor from coconut (*Cocos nucifera* L.) endosperm, changes the seeds oil content in transgenic *Arabidopsis thaliana* and rice (*Oryza sativa* L.). *Frontiers in Plant Science*, ***8*,** p.63.

**Tang, G., Xu, P., Ma, W., Wang, F., Liu, Z., Wan, S. and Shan, L**. 2018. Seed-specific expression of AtLEC1 increased oil content and altered fatty acid composition in seeds of peanut (*Arachis hypogaea* L.). *Frontiers in Plant Science*, ***9***, p.260.

**Van De Loo, F.J., Broun, P., Turner, S. and Somerville, C**. 1995. An oleate 12-hydroxylase from *Ricinus communis* L. is a fatty acyl desaturase homolog. *Proceedings of the National Academy of Sciences*, ***92*(15)**, pp.6743-6747.

**Wan, X., Liu, Q., Dong, B., Pillai, S.V., Huang, F.H., Singh, S.P. and Zhou, X.R**. 2019. Molecular and biochemical analysis of the castor caruncle reveals a set of unique genes involved in oil accumulation in non-seed tissues. *Biotechnology for Biofuels*, ***12*(1)**, pp.1-13.

**Wang, L., Zhang, Y., Li, D., Dossa, K., Wang, M.L., Zhou, R., Yu, J. and Zhang, X**. 2019. Gene expression profiles that shape high and low oil content sesames. *BMC Genetics*, ***20*(1)**, pp.1-11.

**Wang, N., Ma, J., Pei, W., Wu, M., Li, H., Li, X., Yu, S., Zhang, J. and Yu, J**. 2017. A genome-wide analysis of the lysophosphatidate acyltransferase (LPAAT) gene family in cotton: organization, expression, sequence variation, and association with seed oil content and fiber quality. *BMC Genomics*, ***18*(1)**, pp.1-18.

**Webb, H., Lanfear, R., Hamill, J., Foley, W.J. and Külheim, C**. 2013. The yield of essential oils in *Melaleuca alternifolia* (*Myrtaceae*) is regulated through transcript abundance of genes in the MEP pathway. *PLoS One*, ***8*(3)**, p.e60631.

**Wu, X.L., Liu, Z.H., Hu, Z.H. and Huang, R.Z**. 2014. BnWRI1 coordinates fatty acid biosynthesis and photosynthesis pathways during oil accumulation in rapeseed. *Journal of Integrative Plant Biology*, ***56*(6)**, pp.582-593.

**Xiu, Y., Wu, G., Tang, W., Peng, Z., Bu, X., Chao, L., Yin, X., Xiong, J., Zhang, H., Zhao, X. and Ding, J**. 2018. Oil biosynthesis and transcriptome profiles in developing endosperm and oil characteristic analyses in *Paeonia ostii* var. *lishizhenii*. *Journal of Plant Physiology*, ***228***, pp.121-133.

**Xu, R., Wang, R. and Liu, A**. 2011. Expression profiles of genes involved in fatty acid and triacylglycerol synthesis in developing seeds of Jatropha (*Jatropha curcas* L.). *Biomass and Bioenergy*, ***35*(5)**, pp.1683-1692.

**Xu, Z., Li, J., Guo, X., Jin, S. and Zhang, X**. 2016. Metabolic engineering of cottonseed oil biosynthesis pathway via RNA interference. *Scientific Reports*, ***6*(1)**, pp.1-14.

**Yan, B., Xu, X., Gu, Y., Zhao, Y., Zhao, X., He, L., Zhao, C., Li, Z. and Xu, J**. 2018. Genome-wide characterization and expression profiling of diacylglycerol acyltransferase genes from maize. *Genome*, ***61*(10)**, pp.735-743.

**Yang, S., Miao, L., He, J., Zhang, K., Li, Y. and Gai, J**. 2019. Dynamic transcriptome changes related to oil accumulation in developing soybean seeds. *International journal of molecular sciences*, ***20*(9)**, p.2202.

**Yeap, W.C., Lee, F.C., Shabari Shan, D.K., Musa, H., Appleton, D.R. and Kulaveerasingam, H.** 2017. WRI 1‐1, ABI 5, NF‐YA 3 and NF‐YC 2 increase oil biosynthesis in coordination with hormonal signaling during fruit development in oil palm. *The Plant Journal*, ***91*(1)**, pp.97-113.

**Zang X, Geng X, Ma L, Wang N, Pei W, Wu M, Zhang J, Yu J**. 2019. A genome-wide analysis of the phospholipid: diacylglycerol acyltransferase gene family in *Gossypium*. *BMC Genomics*.;**20(1)**:402

**Zhang, C., Ren, H., Yao, X., Wang, K. and Chang, J**. 2021. Full-length Transcriptome Analysis of Pecan (*Carya illinoinensis*) Kernels. *G3-Genes Genom. Genet*., **11**: jkab182.

**Zhang, D., Zhang, H., Hu, Z., Chu, S., Yu, K., Lv, L., Yang, Y., Zhang, X., Chen, X., Kan, G. and Tang, Y**. 2019. Artificial selection on GmOLEO1 contributes to the increase in seed oil during soybean domestication. *PLoS Genetics*, ***15*(7)**, p.e1008267.

**Zhang, F., Gao, X., Zhang, J., Liu, B., Zhang, H., Xue, J. and Li, R**. 2018. Seed-specific expression of heterologous gene DGAT1 increase soybean seed oil content and nutritional quality. *Sheng wu gong cheng xue bao= Chinese Journal of Biotechnology*, ***34*(9)**, pp.1478-1490.

**Zhao, X., Yang, G., Liu, X., Yu, Z. and Peng, S**. 2020. Integrated Analysis of Seed microRNA and mRNA Transcriptome Reveals Important Functional Genes and microRNA-Targets in the Process of Walnut (*Juglans regia*) Seed Oil Accumulation. *International Journal of Molecular Sciences*, ***21*(23)**, p.9093.

**Zhu, D., Le, Y., Zhang, R., Li, X. and Lin, Z**. 2021. A global survey of the gene network and key genes for oil accumulation in cultivated tetraploid cottons. *Plant Biotechnology Journal* **19,** 1170-1182.
